# Expression and Functional Analysis of the BCL2-Associated Agonist of Cell Death (*BAD*) Gene in the Sheep Ovary During the Reproductive Cycle

**DOI:** 10.3389/fendo.2018.00512

**Published:** 2018-09-19

**Authors:** Xiaohan Cao, Xiangyu Wang, Lulu Lu, Xiaoyu Li, Ran Di, Xiaoyun He, Wenping Hu, Xianyin Zeng, Qiuyue Liu, Mingxing Chu

**Affiliations:** ^1^Key Laboratory of Animal Genetics, Breeding and Reproduction of Ministry of Agriculture, Institute of Animal Science, Chinese Academy of Agricultural Sciences, Beijing, China; ^2^Department of Bioengineering, Life Science College, Sichuan Agricultural University, Ya'an, China

**Keywords:** sheep, BAD gene, cell apoptosis, ovary, granulosa cells

## Abstract

Most ewes in China are seasonally polyestrous with normal ovulatory cycles, which is controlled by photoperiod (length of the daily light phase). These ewes are estrous in the short-day season and anestrus in the long-day season and cannot mate during anestrus. Thus seasonal breeding limits both diversification and intensification of production. If sheep can estrus all round year, it can be mated twice per year, which can greatly improve the economic benefits. To change seasonal estrus at the genetic level and cultivating new sheep breeds, it is important to understand the molecular mechanisms of seasonal breeding trait in sheep. The BCL2-associated agonist of cell death (*BAD*) gene being a regulator of cellular apoptosis was identified by our previous RNA-Seq, which is associated with follicular development in mammalian ovaries. However, the mechanism how *BAD* can regulate estrus in sheep was poorly understood. In this study, we characterized ovine *BAD*, including full-length mRNA cloning and protein sequence prediction, as well as *BAD* expression profile in Small-tailed Han (STH) sheep. The highest expression levels of *BAD* were observed in sheep hypothalamus, lung, and pituitary, while the lowest expression was in liver. Functional analysis of *BAD* was performed in primary granulosa cells of sheep. The concentration of P4 was significantly increased after RNAi interference of *BAD*, while P4 level was shown to be opposite after *BAD* overexpression *in vitro*. It has been found that *BAD* can reduce progesterone levels by promoting ovarian GC apoptosis, which might be involved in regulating the estrus cycle in sheep.

## Introduction

Seasonality in reproduction is characteristic of most wild and domesticated species of mammals and birds. Food availability, temperature, and ambient photoperiod (PP; length of the daily light phase) can facilitate or inhibit reproductive function seasonally. The PP serves as the primary factor in the regulation of reproductive timing in long- and short-day breeding mammals and most bird species (1, 2). Upstream signals mainly go through the hypothalamic–pituitary–ovarian axis to regulate gonadotropin and ovarian steroid levels, which in turn influence reproductive activities. Ewes are estrous in the short-day season and anestrus in the long-day season. Seasonal breeding limits both diversification and intensification of production. If sheep can estrus all round year, it might greatly improve the economic benefits.

The ewe is a seasonally polyestrous animal with normal ovulatory cycles. Ovary can secrete estrogen, testosterone and progesterone. As is known, ovarian steroids-mainly estradiol (E2)-can carry out negative feedback actions following gonadotropin-releasing hormone (GnRH) secretion, and the estrogen receptor alpha (ERα) is the predominant mediator of this feedback effect during seasonal breeding. In the mammalian ovary, most follicles undergo selective atresia by a hormonally regulated apoptotic mechanism and only a small fraction of oocytes can ovulate during the animal's reproductive life (3, 4). This is underpinned by the apoptosis of granulosa and theca cells (5). It has been shown that factors secreted from granulosa cells (GCs) such as gonadal steroids, growth factors, and cytokines are essential for follicular growth and atresia during the reproductive cycle and in the transition between the breeding and non-breeding seasons (6).

Apoptosis is a physiological programmed process essential for the elimination of damaged or supernumerary cells (7). There are two best understood apoptotic mechanisms in mammalian cells: the mitochondrial pathway triggered by the activation of proapoptotic BH3 proteins in response to a diverse range of cellular stressors and depends on the release of proteins from the intermembrane space of mitochondria; and the extrinsic pathway, which is activated by extracellular ligands binding to cell surface death receptors (8, 9). Both pathways converge with the activation of caspase proteins, which are responsible for cleaving essential proteins ultimately leading to cellular destruction. The mitochondrial pathway is regulated by the relative levels and activities of both pro- and anti-apoptotic members of the B-cell lymphoma-2 (BCL2) superfamily. These proteins share one or more of the four BCL2 homology domains (BH1–BH4) that can be divided into multiple subgroups. The pro-apoptotic BH3-only proteins such as the BCL2 antagonist of cell death (BAD), BH3-interacting domain death agonist (BID), BCL2-interacting mediator of death (BIM), NOXA, and p53 upregulated modulator of apoptosis (PUMA) share the single BH3 domain.

The BAD protein was first identified in the rat ovary by a yeast two-hybrid system being an ovarian BCL2-interacting protein and was one of the first pro-apoptotic proteins shown to have a role in follicular atresia (10). Active (unphosphorylated) BAD is mediated through its binding and neutralizing BCL2, BCL-X_L_, and BCL-W (11, 12). The addition of survival factors correlated with the phosphorylation of BAD on positions Ser-112,-136, and-155, which results in its binding to 14-3-3 proteins in an inactive state (13, 14).

*Bad*^−/−^ gene knockout mice have excess blood platelets and progress to B cell lymphoma with aging (15). Rat Bad mRNA is expressed in GCs of different sized follicles and in theca cells, and *Bad* gene overexpression can induce apoptosis in both cell types (16).

Here, we characterized the ovine *BAD* gene, including its complete cDNA sequence, predicted protein sequence and expression profiling in Small-tailed Han (STH) sheep. Furthermore, functional analysis of the *BAD* gene was performed in primary sheep GCs using RNA interference (RNAi) and lentivirus-mediated overexpression techniques. We found that *BAD* is a candidate gene that might initiate the estrus cycle in sheep via ovarian cell apoptosis.

## Materials and methods

### Experimental animals and sample collection

All procedures that involved animals were approved by the Animal Care and Use Committee of Chinese Academy of Agricultural Sciences, Beijing, P. R. China and the Animal Care and Use Committee of Ningxia Academy of Agriculture and Forestry Sciences, Yinchuan, Ningxia, P. R. China. All animal tissue collection procedures were as described (17). Briefly, healthy non-pregnant 3-year-old Tan and STH ewes were selected from respective breed conservation farms and housed in the same farm in Ningxia Autonomous Region, P. R. China. The ewes were examined daily for estrous activity with a teaser ram during all four seasons. The dates of estrous cycles and duration of estrus were recorded and blood was collected daily for measurement of serum hormone concentrations as described (18–20). Estrus was judged based on obvious behavioral signs in response to the teaser ram. The healthy non-pregnant 3-year-old Tan and STH ewes were selected for expression profile of BAD gene. Three ewes were selected arbitrarily and euthanized for tissue collection including the hypothalamus, pituitary, ovary, uterus, heart, liver, spleen, lung, and kidney. All tissue samples were immediately snap-frozen in liquid nitrogen for future use.

### Cell culture and immunofluorescence staining

The age of sheep used in cell culture experiment was 3–5 years old, and cycle stage of sheep used in cell culture experiment was follicular phase. Fresh ovaries were harvested from healthy adult female STH sheep in slaughterhouses.

Ovaries were taken back to the laboratory in phosphate-buffered saline (PBS) at 4°C. They were washed with 75% ethanol twice (5–10 s each time) and then with sterile saline three times to remove the alcohol. Connective tissues were removed carefully. Follicular fluid samples containing GCs were aspirated by syringe from visible follicles (>3.0 mm in diameter). The GCs were separated from the follicular fluid by centrifugation for 5 min at 1,500 rpm followed by washing with sterile Dulbecco's Modified Eagle's Medium (DMEM; high glucose, HyClone, Logan, UT, USA) two times. The cells were evenly plated onto cell culture plates (Costar, Cambridge, MA, USA) at a density of 10^5^ cells/cm^2^ in the same medium supplemented with 15% fetal bovine serum (FBS; Gibco, Waltham, MA, USA) and 1% penicillin-streptomycin solution (Gibco) and then incubated at 37°C under 5% CO_2_ in humidified air.

Immunofluorescence staining was used to identify follicle stimulating hormone receptor (FSHR) specifically expressed in granulosa cells. After fixed cells with 4% polyoxymethylene and washed with PBS, added 10% Donkey Serum in 0.1% PBS/ Triton-X, blocking 20 min. An antibody (FSHR 1:500. BAD 1:500) was incubated with 8% Donkey Serum in 0.1% PBS/ Triton-X at 4C° for 12 h, followed by washing in PBS three times. Then, diluted second antibody (1:1,000) was incubated at room temperature for 1 h in dark, followed by washing with PBS three times. Add 15 μL ProLong antifade reagent and Hoechst mixture on the fixed-cell surface and cover glass slide carefully, preserved in the dark at 4C° until observed.

### Total RNA isolation and cDNA preparation

RNA from the GCs was extracted with TRIzol solution (Invitrogen, Thermo Fisher Scientific Inc., Waltham, MA, USA) and digested with DNase and adsorption columns (RNAprep pure Micro Kit DP420, Tiangen Biotech., Beijing, P. R. China) to remove possible DNA contamination. The quality and concentration of extracted RNA was examined by 1% agarose gel electrophoresis and a NanoDrop 2000 (Thermo Fisher Scientific Inc.). cDNA for gene amplification and expression was synthesized using a PrimeScript® RT Reagent Kit (RR037A, Takara, Dalian, P. R. China).

### Molecular cloning of *BAD* by 3′ and 5′ rapid-amplification of cDNA ends (RACE) and sequence analysis

The primers for the molecular cloning shown in Table 1 were designed according to the *Ovis aries BAD* sequence (GenBank ID: XM_004019650). After the partial coding DNA sequence (CDS) had been cloned, gene-specific primer (GSP) sequences for 3′ and 5′ RACE were designed to obtain the 3′ and 5′ UTR of the *BAD* gene. The PCR amplifications were performed using the following protocol: initial denaturation for 3 min at 94°C, followed by 12 cycles of denaturation for 30 s at 94°C, annealing for 30 s at 75°C (with a decrease of 0.5°C per cycle), and extension for 3 min at 72°C; another 25 cycles of 30 s at 94°C, 30 s at 68°C, and 3 min at 72°C, and a final extension for 8 min at 72°C. The PCR products were purified using DNA Purification Kits (TIANGEN Biotech Co., Ltd., Beijing, P. R. China) and cloned into the pMDTM18- T Vector (Takara Bio Inc., Dalian, P. R. China). Positive clones were sent for commercial sequencing (Sangon, Shanghai, P. R. China).

**Table 1:** Primers for the molecular cloning of *BAD* gene and *BAD*-siRNA sequences.

**Purpose**	**Name of sequences**	**Sequence(5′ → 3′)**
Molecular cloning of *BAD* gene	*BAD*-F	CAGGGGCCTCGTTATCGG
	*BAD*-R	GGACTCTGGATCAGACCTCA
RNA interference of *BAD* gene	*BAD*-siRNA001	Positive-sense strand: 5′GCAACGCAAAUGCGACAAA dTdT 3′
		Antisense strand: 3′dTdT CGUUGCGUUUACGCUGUUU 5′
	*BAD*-siRNA002	positive-sense strand: 5′GGCUCGGAUUCGUUCCUUU dTdT 3′
		Antisense strand: 3′dTdT CCGAGCCUAAGCAAGGAAA 5′
	*BAD-*siRNA003	Positive-sense strand: 5′CUCAGCAAGCACUGGCUAA dTdT 3′
		antisense strand: 3′dTdT GAGUCGUUCGUGACCGAUU 5′
qRT-PCR of *BAD* gene	*qPCR-BAD-F*	CAGGGGCCTCGTTATCGG
	*qPCR-BAD-R*	GGACTCTGGATCAGACCTCA
	*18S-F*	CGGCTACCACATCCAAGGAA
	*18S-R*	GCTGGAATTACCGCGGCT

### *BAD* and steroidgenic genes expression analysis by RT-qPCR

The expression profile of *BAD* in STH sheep was detected by RT-qPCR; *18s* rRNA was adopted as an internal control. The primers for *BAD* and *18s* rRNA were designed using primer3 (v.0.4.0; http://bioinfo.ut.ee/primer3-0.4.0) and the sequences are shown in Table 1. Four steroidogenic genes expression detected by qRT-PCR. The information of the four genes (*3*β*-HSD, 17* β*-HSD, p450scc*, and *CYP11*) shown in Table 2. Each 20 μL PCR reaction mix contained 10 μL KAPA SYBR FAST master mix (KAPA Biosystems, Woburn, MA, USA), 2 μL cDNA, 0.4 μL forward primer, and 0.4 μL reverse primer, and the rest of the volume was supplemented with double-distilled (dd) H_2_O. Each qPCR run was performed at 95°C for 10 min and then for 40 cycles at 95°C for 30 s, 60°C for 20 s using KAPA SYBR® FAST qPCR kits (KAPA Biosystems,) in the Roche LightCycler® 480 II RT-PCR system (Roche Applied Science, Branford, CT, USA). All the reactions were performed in triplicate. Non-template reactions (replacing cDNA with RNase-free H_2_O) were used as negative controls.

**Table 2:** Primers for the four steroidgenic genes sequences.

**Name of sequences**	**Sequence(5^′^ → 3^′^)**
***3**β**-HSD-F***	GGAGACATTCTGGATGAGCAG
***3**β**-HSD-R***	TCTATGGTGCTGGTGTGGA
***17** β**-HSD-F***	GTAGGGTTGCTGGTTTGCCT
***17** β**-HSD-R***	TCCCAATCCCATCTCCTGCT
***p450scc-F***	AGACGCTAAGACTCCACCCT
***p450scc-R***	CCACCTGGTTGGGTCAAACT
***CYP11-F***	GTTTCGCTTTGCCTTTGAGTC
***CYP11-R***	ACAGTTCTGGAGGGAGGTTGA

### RNA silencing

*BAD* siRNAs were synthesized by Guangzhou Ribobio Co., Ltd., (Guanzhou, P. R. China) and the sequences are shown in Table 1. All experiments were performed in triplicate. The GCs were seeded in six-well plates at a density of 3 × 10^5^ cells/well in DMEM supplemented with 15% FBS and incubated at 37°C in 5% CO_2_ in humidified air. Cells were subjected to transfection when they reached 70–90% confluency. *BAD* siRNA and Lipo3000 were diluted using Opti-MEM according to the manufacturer's manual, and the mixture was added to cultured cells. After incubating for 6 h, the medium was replaced with fresh serum-free DMEM for 2–4 days extra. Then, cells were collected for RNA and protein extraction, and the supernatant was collected for E2 and P4 detection. For selecting an optimal transfection concentration of siRNA, 3 targeting siRNAs and 2 different transfection concentration, 50 and 30 nM, were chosen for testing BAD-siRNA efficiency (Supplemental Figure 1). For preventing non-specificity, non-targeting control siRNA was designed to make sure siRNA specificity of our targeting siRNA, and blank control was used as the transfection control.

### Concentration of P4 and E2 hormone

Concentrations of P4 and E2 were detected by radioimmunoassays in Beijing North Institute of biological Technology.

### Lentivirus-mediated overexpression of *BAD* gene

Sheep GC lines with stable *BAD* overexpression were established using a lentiviral expression system. Briefly, the *BAD* CDS region (Lentivirus-mediated *BAD*, LV-*BAD*) or a negative control (LV-NC) were cloned into the GV365 transfer vector (GeneChem Company, Shanghai, P. R. China) by AgeI/AgeI enzyme digestion. A lentiviral packaging system and transfer vector were used to perform cotransfection in a 293T cell line (293T cell line is derived from 293 cells which a human renal epithelial cell line transfected with adenovirus E1A gene, and express SV40 large T antigen.) to get high titers of lentiviral particles according to the instruction manual (GeneChem Co., Shanghai, P. R. China). The transfer vector expressed EGFP and the ampicillin resistance gene, which allowed for measuring the infection efficiency by flow cytometry. Sheep GCs at a density of 5 × 10^8^ cells/mL were infected with LV-*BAD*, LV-NC or virus-free medium in DMEM supplemented with 15% FBS and 5 μg/mL polybrene (GeneChem Co.). The concentration of vector-BAD was 100MOI. Cells were treated for 12 h, then the medium was replaced with DMEM with 15% FBS. Cells were harvested using 0.05% trypsin-EDTA (# 25300-054, Gibco) and resuspended in culture medium to be sorted by flow cytometry (Beckman Moflo XDP, Beckman Coulter, Inc., Fullerton, CA, USA). The EGFP-positive cells were cultured for further analysis.

### Western blotting

Protein extracts were prepared by complete homogenization of cells in immunoprecipitation buffer (Beyotime, Shanghai, China) according to the manufacturer's instructions. Equal amounts of protein extracts were mixed with sample buffer and separated by SDS–PAGE. Separated proteins were transferred electrophoretically to PVDF (polyvinylidene fluoride) membranes (MDBio Inc., Shandong, P. R. China). Blots were incubated with 10 mL 5% skim milk powder in Tris-buffered saline with Tween-20 (TBST) overnight at 4°C. An anti-BAD protein antibody (Abcam, Cambridge UK. 1:10,000) was incubated with PVDF membranes at room temperature for 2 h, followed by washing in TBST four times, 10 min each. Then, diluted Goat anti-Rabbit IgG (horseradish peroxidase labeled, HRP, Abcam, UK, 1:10,000) was incubated at room temperature for 2 h, followed by washing in TBST four times, 10 min each. Stock solutions of 20 × LumiGLO and 20 × hydrogen peroxide were diluted to 1 × with ddH_2_O, added dropwise to the PVDF membranes and incubated in the dark for 1 min, grabbing image by Molecular Imager® Gel Doc™ XR System (BIO-RAD, USA) in 30 min.

### Cell apoptosis assays

Apoptosis was examined by TUNEL assays. GCs (about 1 × 10^5^ cells/cm^2^) were cultured in 12-well plates and fixed with 4% paraformaldehyde for 1 h at room temperature. Cells were washed and stained using *in situ* Cell Death Detection kits, POD (11684817910, Roche Diagnostics) according to the manufacturer's instructions. Apoptosis was observed by fluorescence microscopy (DMIL LED Fluo 374625, Leica Microsystems, Bannockburn, IL, USA). Annexin V-FITC Apoptosis Detection kits (K101; Bio-Vision Inc., Mountain View, CA, USA) were used for measuring apoptotic cell death in a dual-staining protocol according to the manufacturer's instructions. In this system, apoptotic cells are stained with Annexin V-FITC (green fluorescence) while necrotic cells are stained with PI (red fluorescence). GCs (about 5 × 10^5^ cells) were collected in binding buffer, and stained with 5 μL of Annexin V-FITC and 5 μL of PI at room temperature for 5 min in the dark. Stained cells were immediately analyzed by flow cytometry (Beckman Coulter Inc., Miami, FL, USA; excitation wavelength 488 nm; emission wavelength 530 nm) using an FITC signal detector (FL1) and a phycoerythrin emission signal detector (FL2). Each experiment was done in triplicate.

### Statistical analysis

Duncan's multiple range test was used to calculate standard errors among replicated samples using IBM SPSS Statistics (v. 24.0; IBM Corp., Armonk, NY, USA). The 2^−ΔΔ*CT*^ method was used to calculate relative mRNA levels (24). Analysis of variance (ANOVA) was used to examine significant differences of expression levels between samples with SAS v. 9.2 (SAS Institute Inc., Cary, NC, USA). One-way ANOVA was used to analyze the flow cytometry data (Summit 5.2.0 software; http://summit1.software.informer.com/5.2/).

## Results

### Results of solexa sequencing and expression profile of ovine *BAD*

**Figure 1:**
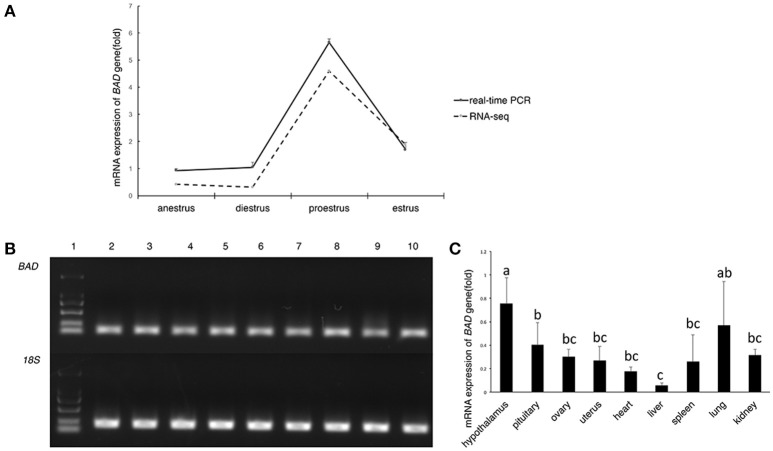
Expression profiling of *BAD* gene **(A–C)**. **(A)** *BAD* gene expression in sheep ovaries at different stage of oestrous cycle by RNA-seq and real-time PCR; **(B)** Expression level of *BAD* gene analyzed by semi—quantitative PCR. 1: Marker; 2-10: uterus, ovary, pituitary, hypothalamus, kidney, lung, spleen, liver, heart; *BAD*: *BAD* gene; 18S: *18S ribosomal RNA* gene. **(C)** Relative expression level of *BAD* gene in different tissues of Small Tail Han sheep. Lowercase letters means significant differences (*P* < 0.05).

Among the differentially expressed genes detected by RNA-sequencing (seq) during anestrus and the breeding season (unpublished data, the RNA-sequencing data reported herein have been deposited to the Genome Sequence Archive (http://gsa.big.ac.cn) of the BIG Data Center 34 under accession number PRJCA000881.), *BAD* expression was significantly upregulated during proestrus (*p* < 0.01; Figure 1A) in STH sheep. To validate the results of RNA-seq results of STH and Tan ewes, *BAD* gene expression levels in the ovary during different reproductive stages of the STH ewes were measured by quantitative reverse transcription polymerase chain reaction (RT-qPCR) amplification. As shown in Figure 1A, *BAD* was significantly upregulated (*p* < 0.05) during proestrus compared with other stages, which was consistent with the RNA-seq data. The distribution of *BAD* mRNA expression among tissues (heart, liver, spleen, lung, kidney, hypothalamus, pituitary, uterus, and ovary) was investigated. The highest expression levels of *BAD* measured by RT-qPCR were observed in the hypothalamus, lung and pituitary. The lowest expression was in the liver (Figures 1B,C).

### Molecular cloning of the complete cDNA sequence of the *BAD* gene in STH sheep

**Figure 2:**
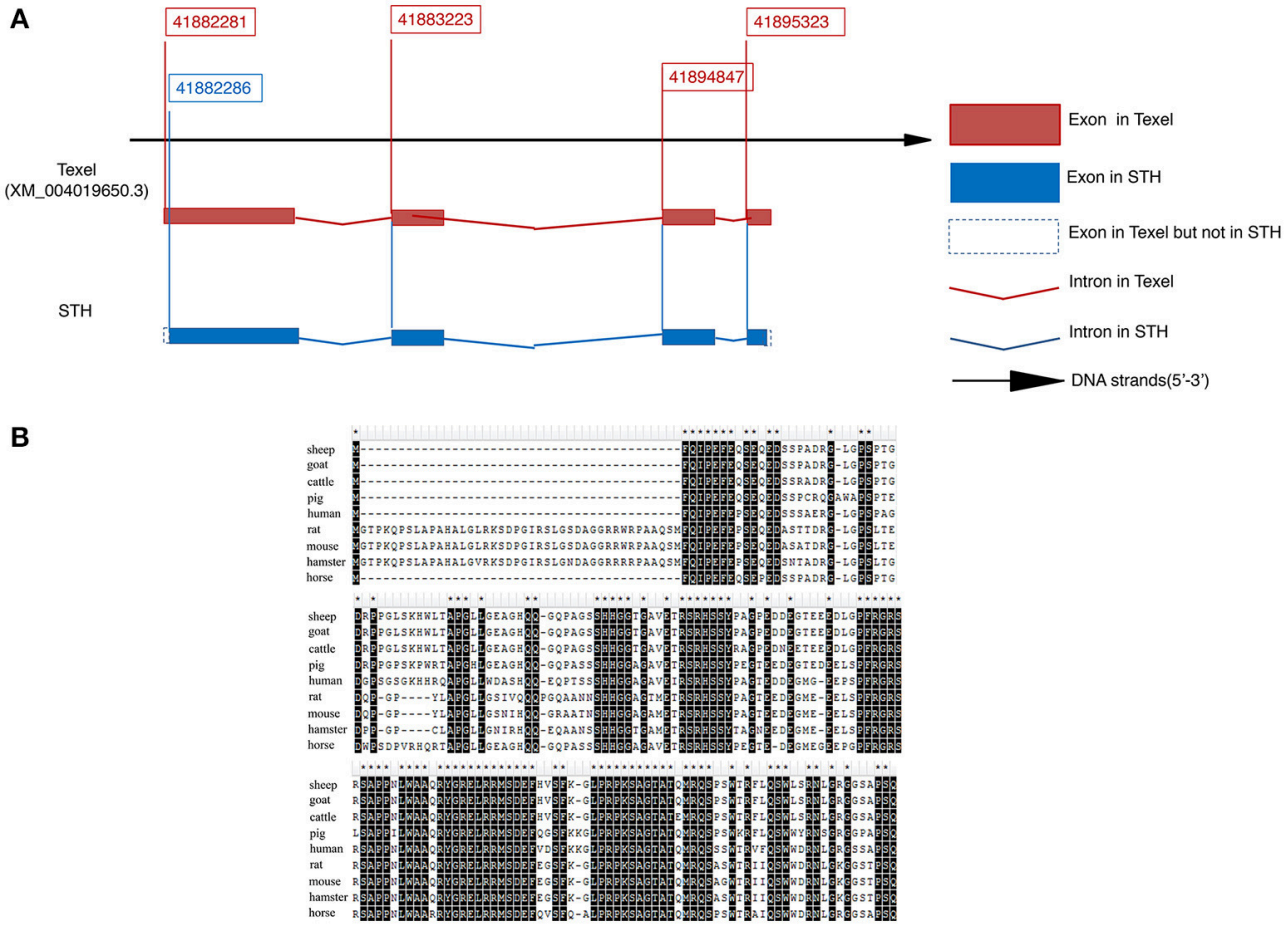
Molecular cloning and characterization of *ovine BAD* gene in Small Tail Han sheep **(A,B). (A)** mRNA sequence of *BAD* gene. **(B)** protein sequence of *BAD* gene. Multiple alignment of sheep BAD protein sequence with orthologs from other species (sheep, goat, cattle, pig, human, rat, mouse, hamster and horse).

We used 5′ and 3′ random amplification of cDNA ends (RACE) to identify the full-length mRNA of the *BAD* gene. A 307-bp 5′ RACE amplicon and a 666-bp 3′ RACE amplicon were obtained by PCR. The *BAD* transcript of STH sheep contains 948 bp, including a 507-bp open reading frame, an 81-bp 5′ untranslated region (UTR) and a 462-bp 3′ UTR (GenBank accession no. KY320499). As shown in Figure 2A, STH and Texel sheep have different transcription start sites (TSS); thus, the TSS of *BAD* in Texel is 30 bp further upstream than in STH sheep. Alignment analysis indicated that the *BAD* gene contains four exons and three introns in the STH genome. The predicted ovine BAD protein is composed of 168 amino acids, and its molecular weight, as predicted by ExPASy Compute pI/Mw tool (http://au.expasy.org/tools/pi_tool.html), is 18.26 kDa, with an isoelectric point of 6.60. Multiple alignment of the sheep BAD protein sequence with orthologs from other species (sheep, goat, cattle, pig, human, rat, mouse, hamster, and horse) indicated a high level of amino acid identity among functional motifs (Figure 2B).

### Steroidgenic genes expression after *BAD* silencing and overexpression

**Figure 3:**
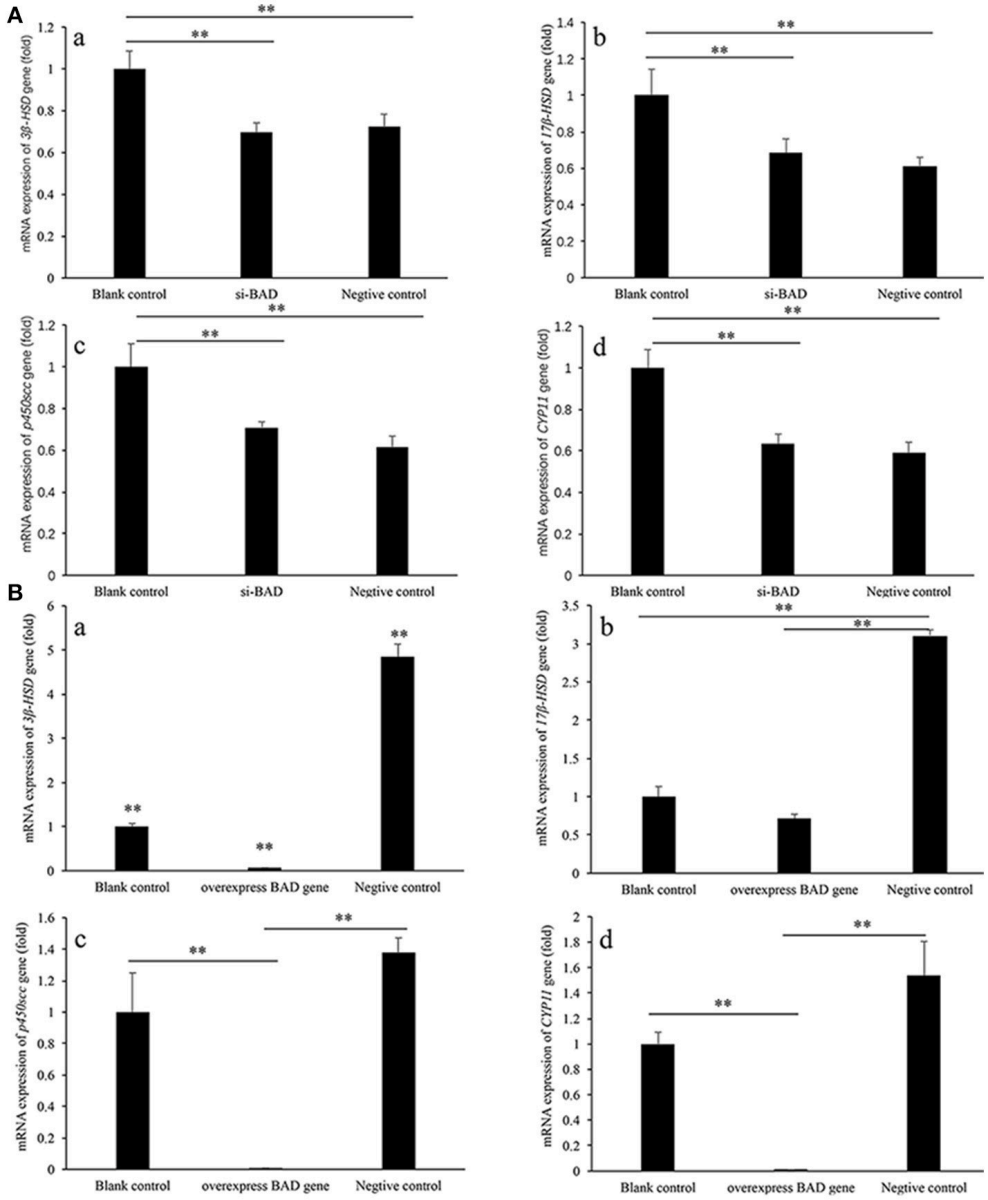
*3*β*-HSD, 17* β*-HSD, p450scc*, and *CPY11* gene expression levels in granulose cells after siRNA interference **(A)** and overexpression **(B)** of *BAD*. **(A)** The expression levels of *3*β*-HSD, 17* β*-HSD, p450scc*, and *CYP11* genes after siRNA interference of *BAD* were detected by real-time PCR. **(B)** The expression levels of *3*β*-HSD, 17* β*-HSD, p450scc*, and *CYP11* genes in *BAD*-overexpressed granulose cells were detected by real-time PCR. ^**^Significant differences (*P* < 0.01).

In siRNA transfection experiment groups, all these four genes showed similar expression characteristics. The expressions of *3*β*-HSD, 17* β*-HSD, p450scc*, and *CYP11* genes in blank control group were significantly higher than those in over-*BAD* group and negative control group (*P* < 0.01), between si-*BAD* group and negative control group, there is no significantly difference. (Figure 3A).

In *BAD*-overexpress experiment groups, all these four genes showed the similar expression characteristics, too. The expressions of *3*β*-HSD, 17* β*-HSD, p450scc*, and *CYP11* genes in negative control group were significantly higher than those in overexpress *BAD* group and blank control group (*P* < 0.01). The expressions of *3*β*-HSD, p450scc*, and *CYP11* genes in overexpress*-BAD* group were significantly lower than those in blank control group (*P* < 0.01). Expression level of *17* β*-HSD* gene had no significantly difference between blank control group and overexpress *BAD* group (Figure 3B).

### RNAi of *BAD* gene in sheep GCs

**Figure 4:**
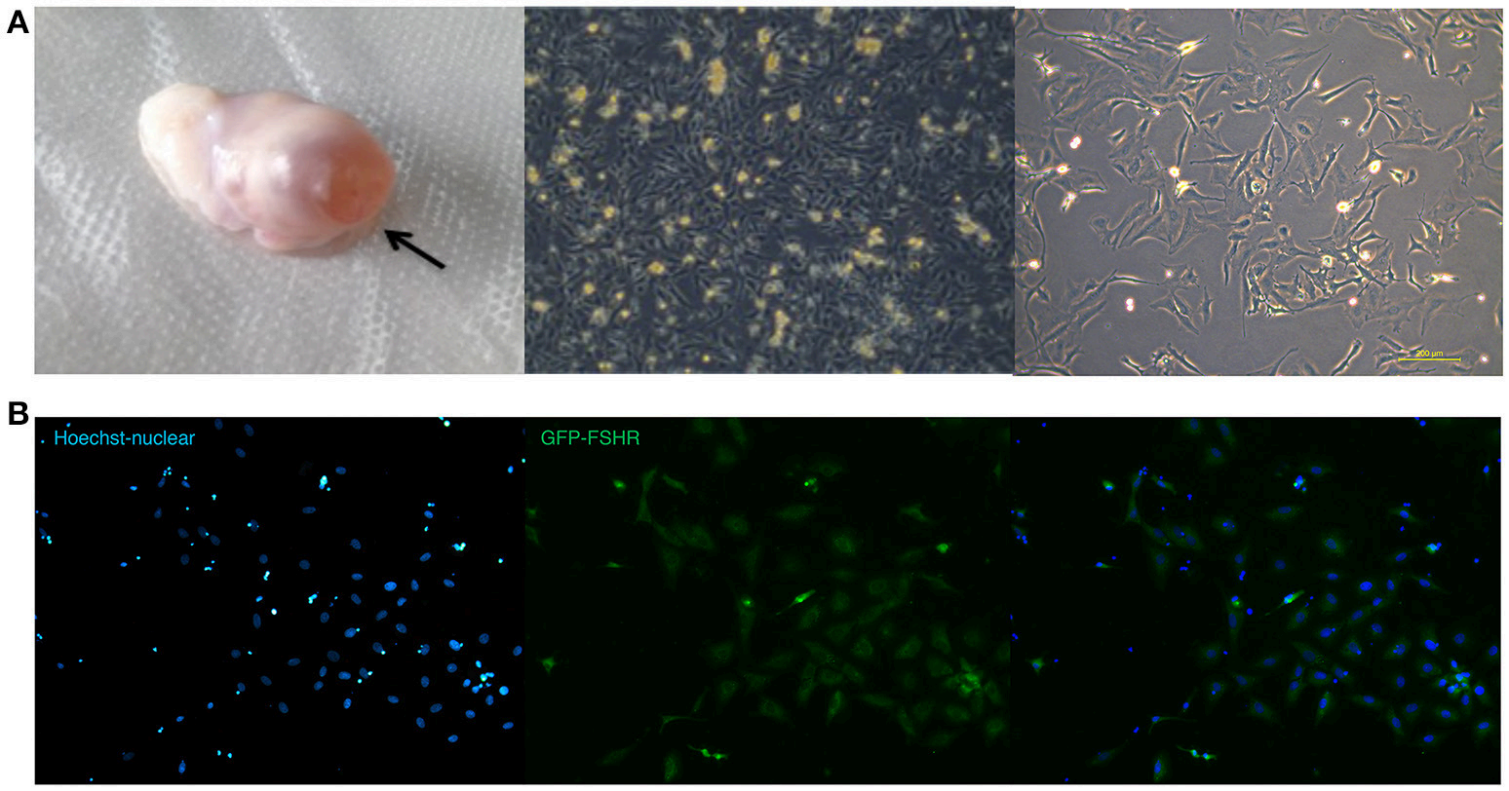
Ovarian granulosa cells of sheep **(A,B)**. **(A)** Ovary with follicle at pre-ovulation stage and images of sheep granulosa cells separated from follicles. **(B)** FSHR expression in sheep granulose cells.

GCs derived from ovaries of STH sheep were spindle-shaped cells and grew as an adherent cell monolayer (Figure 4A). As shown in Figure 4B, the purity of primary GCs was very high, with >90% of GCs expressing the follicle stimulating hormone receptor (FSHR) at 48 h after isolation, as shown by immunofluorescence staining.

**Figure 5:**
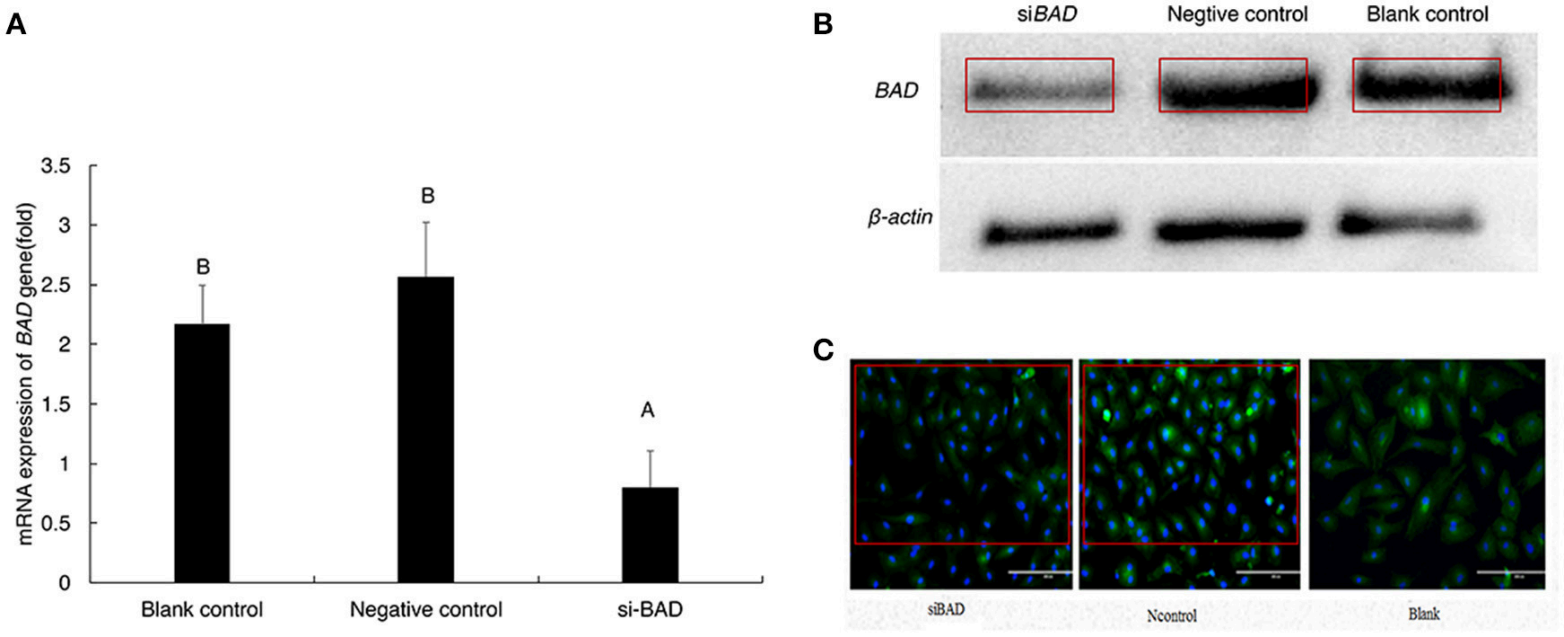
mRNA and protein expression of *BAD* gene after transfection **(A–C)**. **(A)** mRNA expression level of *BAD* gene was detected by real-time PCR after transfection. **(B)** BAD expression at protein level was detected by Western-blot after transfection. **(C)** BAD-positive cells detected by cell Immunofluorescence after transfection(200X). siBAD, interference group; Ncontrol, Negative control group; Blank, Blank control group. Uppercase letters means significant differences (*P* < 0.01).

**Figure 6:**
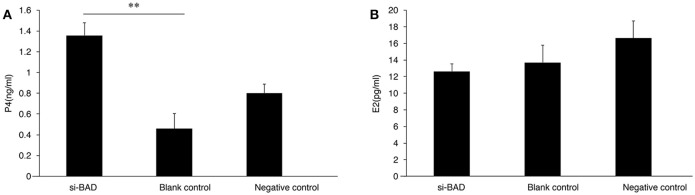
P4 **(A)** and E2 **(B)** levels secreted by granulose cells after transfection were detected by radioimmunoassay **(A,B)**. **(A)** Progesterone concentrations in interference group, blank control group, and negative control group. **(B)** Estradiol concentrations interference group, blank control group, and negative control group. Blank, Blank control group; Ncontrol, Negative control group; siBAD, interference group.

To study whether changes in *BAD* expression levels could affect gonadal steroid secretion levels, which are essential for follicular growth and atresia during the estrous cycle, RNAi was performed on primary GCs. Firstly, the 3 *BAD*-siRNA molecules were transfected in the follicle GCs cultured in the experiment. *BAD*-siRNA002 was selected as interfering molecule to perform experiments in granulosa cells, and the transfection concentration of siRNA002 was 30 nM. As shown in Figure 5A, after silencing of *BAD* expression by siRNA for 48 h the cells showed significantly decreased *BAD* gene expression levels compared with control groups (*p* < 0.01). Furthermore, BAD protein levels were detected by western blotting and immunofluorescence staining after RNAi, and the levels and numbers of BAD-positive cells in the RNAi group were obviously inhibited or decreased (*p* < 0.01) after transfection (Figures 5B,C). The interference efficiency of *BAD* siRNA designed in this experiment was high. Results also revealed that *BAD* was mainly expressed in the cytoplasm of GCs, with low expression in cell membranes. After incubation of siRNA-transfected GCs for 48 h, the concentrations of progesterone (P4) and E2 secreted into the culture medium were detected by radioimmunoassays. As shown in Figure 6, the concentration of progesterone in the RNAi group was increased significantly (*p* < 0.01) while the level of E2 secreted by GCs was decreased slightly but not significantly (*P* > 0.05) compared with control groups. RNAi results showed that the downregulation of *BAD* gene expression had no significant effects on the secretion of E2 but could induce P4 production in GCs.

### Overexpression of *BAD* in sheep GCs induced apoptosis

**Figure 7:**
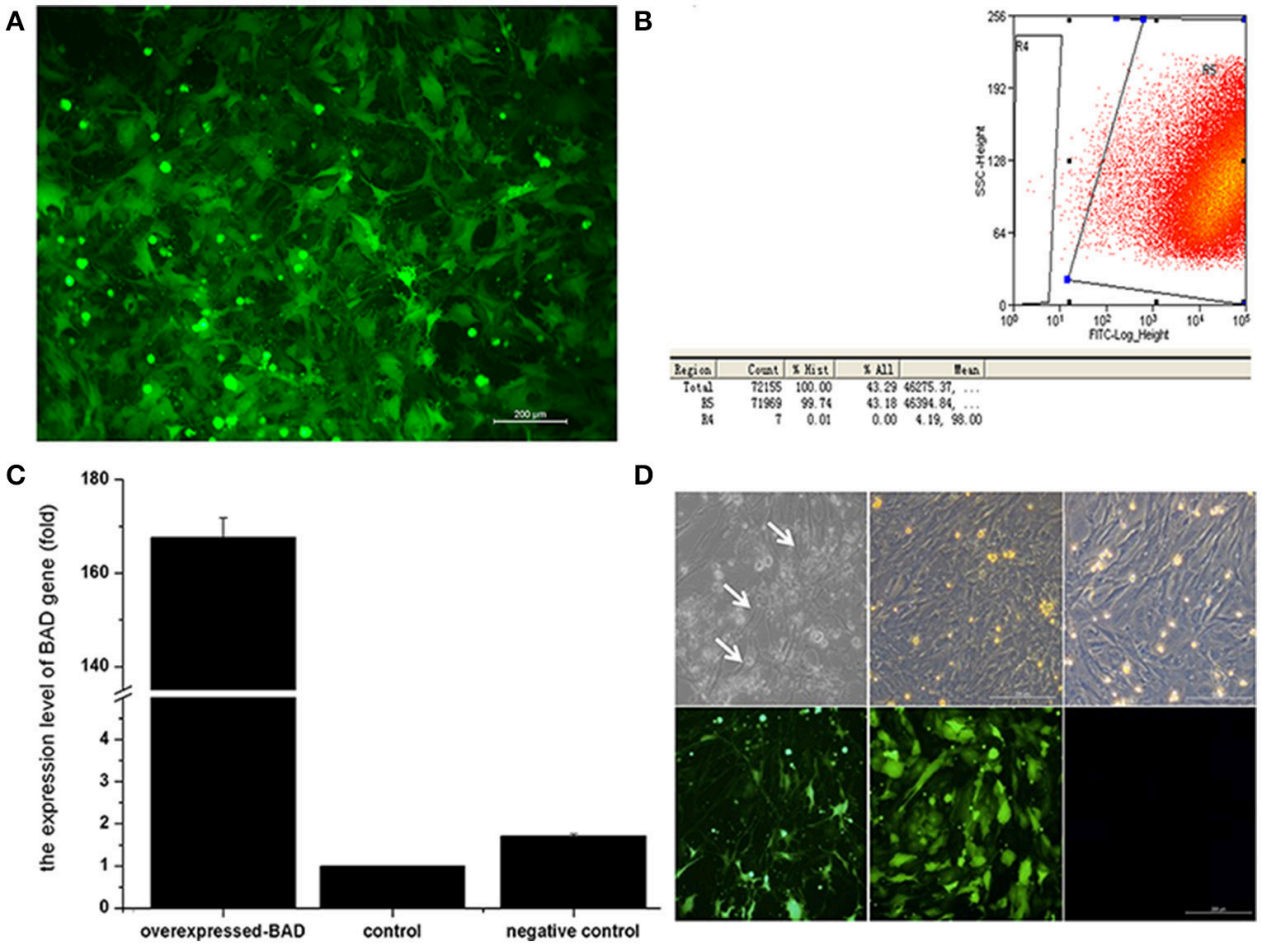
Overexpression of *Bad* gene in ovarian granulosa cells **(A–D)**. Effects of BAD on the cell apoptosis and gonadal steroids secretion in primary sheep granulosa cells were investigated. **(A)** Fluorescence image of granulosa cells after lentiviral particles infection. **(B)** Flow cytometry sorting results of GFP-positive cells. **(C)** mRNA expression of *BAD* gene among blank control, negative control and overexpressed groups. **(D)** Cell images of overexpressed groups, negative control and blank control under light and fluorescence microscopy, respectively. Arrows indicates the apoptotic cells arrows indicates the apoptotic cells.

Given the known link between *BAD* expression and cell apoptosis (25), we further investigated the effect of *BAD* on apoptosis and the secretion of gonadal steroids in primary sheep GCs. After lentiviral infection, GCs were collected and subjected to fluorescent activated cell sorting (FACS) using an enhanced green fluorescent protein (EGFP) marker. As seen in Figures 7A,B, ~99% of GCs were expressing EGFP 72 h after infection as assessed by FACS analysis. Thus, a stable *BAD*-overexpression GC line was established whose expression was 100-fold higher than in control s (Figures 7C,D).

BAD was one of the first pro-apoptotic proteins shown to have a role in follicular atresia. Active (unphosphorylated) BAD is mediated by binding and neutralizing BCL2, BCL-XL, and BCL-W. Moreover, *BAD* expression is significantly upregulated during the transition from anestrus to estrus (16, 26). Therefore, we speculated that *BAD* might function via initiation of apoptosis to alter hormonal secretion in the sheep ovary, and thereby play a role in the transition of seasonal breeding. To test this, expressions of *BAD* gene and apoptosis-related genes (*BAX* and *Bcl-2*) were traced during the differentiation of sheep GCs in our *BAD*-overexpressing cell line mentioned above.

**Figure 8:**
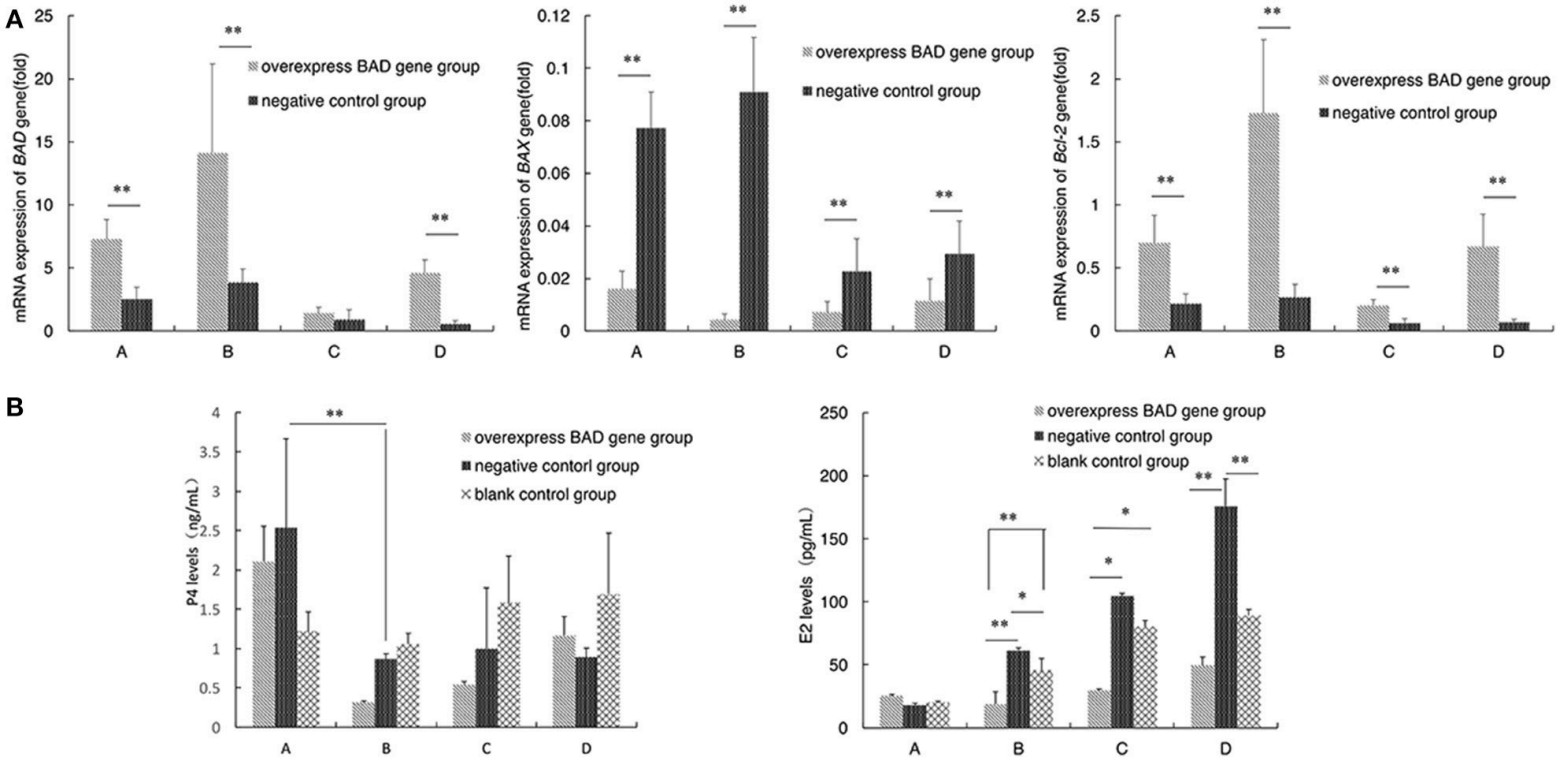
Apoptosis - related genes expression and the P4 and E2 levels secreted by granulose cells after overexpressed **(A,B)**. Expressions of *BAD* gene and apoptosis-related genes (*BAX* and *Bcl-2*) were traced during the differentiation of sheep granulosa cells in our *BAD*-overexpression cell line. mRNA levels of three genes were detected at 4 time points (5, 7, 11, and 13 days) after virus infection. **(A)** Relative expression levels of *BAD*, BAX, and *Bcl-2* genes at 4 time points. A:5 days; B:7 days; C:11 days; D:13 days; D **(B)** The concentrations of progesterone (P4) and estradiol (E2) secreted into cultured medium were detected by radioimmunoassay. ^*^Significant differences (*P* < 0.05). ^**^Significant differences (*P* < 0.01).

The mRNA levels of three genes were detected at four time points (5, 7, 11, and 13 days) after virus infection. The relative expression levels of *BAD* and *Bcl-2* decreased continuously with the differentiation of GCs, and the mRNA expression levels of *BAD* and *Bcl-2* in the overexpressing cells were both higher than in control cells at each point. However, *BAX* mRNA expression displayed an inverted relationship to *Bcl-2* whose expression in the overexpressing group was significantly lower than in the negative control group (Figure 8A).

The concentrations of P4 and estradiol E2 secreted into the culture medium were also detected by radioimmunoassay after virus infection. As shown in Figure 8B, the concentration of P4 in the overexpressing group decreased significantly (*p* < 0.01) at day 7 compared with day 5 after infection. After that, the P4 levels increased gradually in the overexpressing group, while the P4 levels of negative control group were stable after day 7. The concentration of E2 was increased with the differentiation of sheep GCs among all groups after infection. Interestingly, the concentration of E2 in the overexpressing group was always the lowest among three groups at each time point after 5 days since infection.

**Figure 9:**
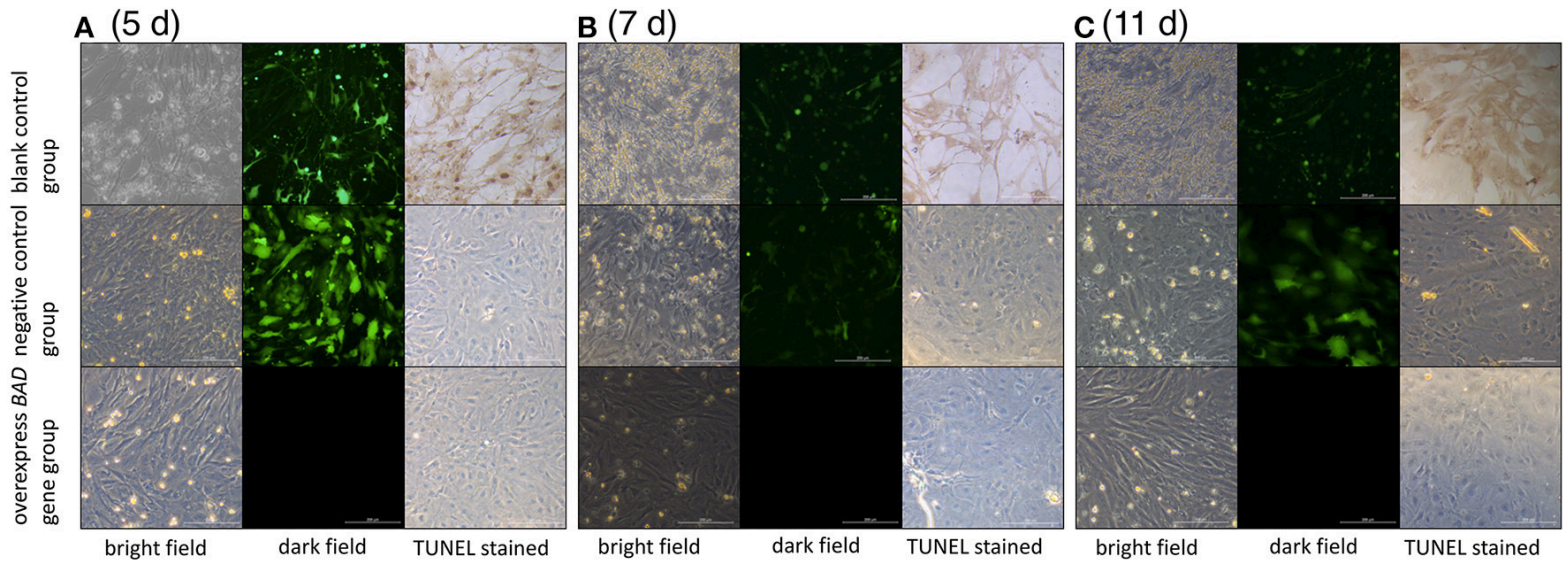
Cell morphologies under light and fluorescence microscopy after TUNEL staining The cell morphology among blank control, negative control, and overexpressed groups under light and fluorescence microscopy after TUNEL staining at **(A)** day 5, **(B)** day 7, and **(C)** day 11, respectively.

**Figure 10:**
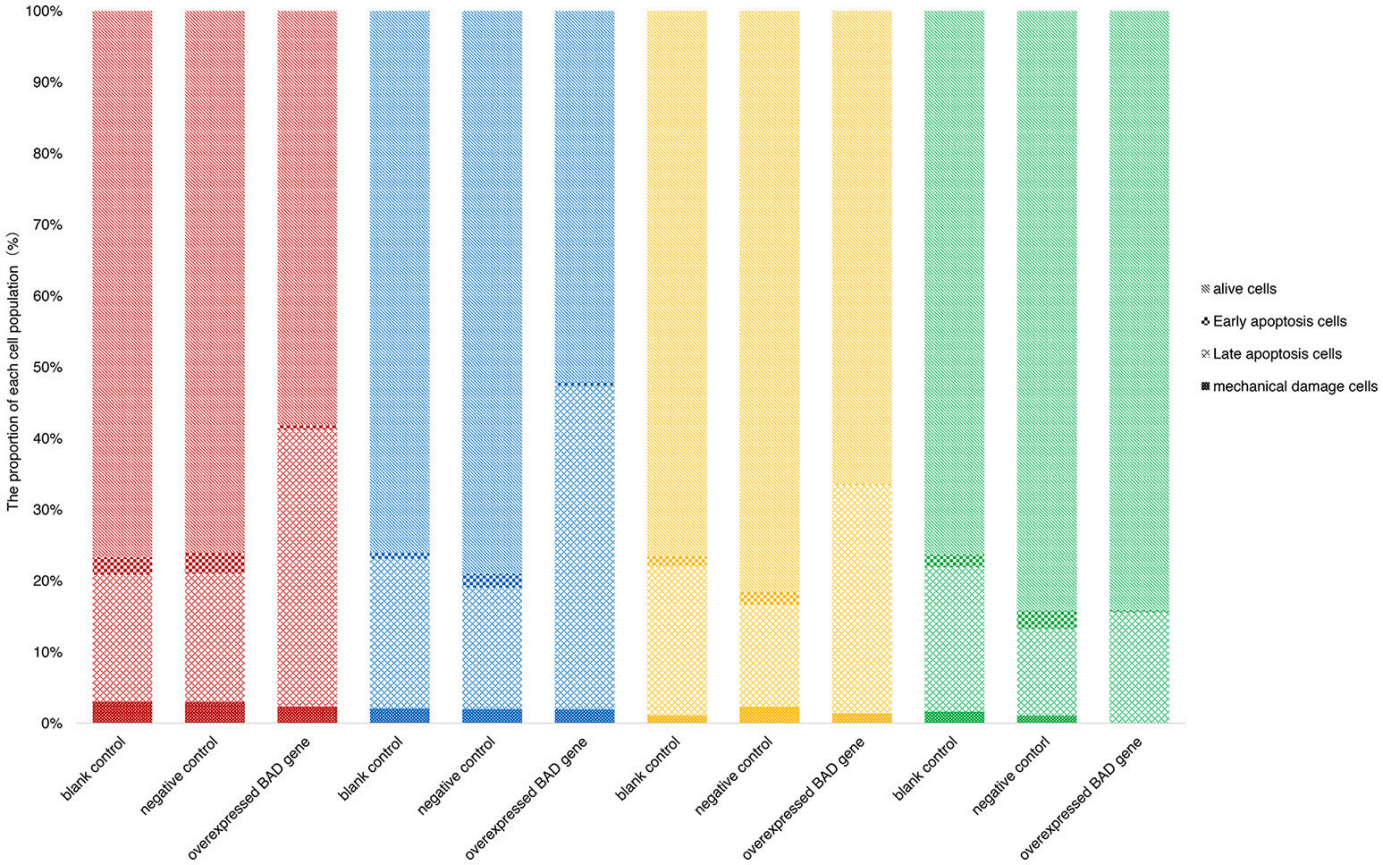
Percentages of early apoptotic, late apoptotic and necrotic cells in blank control, negative control, and overexpressed groups. Annexin FITC/PI flow cytometric analysis was performed using our *BAD*-overexpressed cell line at the 5th (red), 7th (blue), 11th (yellow), and 13th (green) days after infection.

Terminal deoxynucleotidyl dUTP nick end labeling (terminal dexynucleotidyl transferase(TdT)-mediated dUTP nick end labeling, TUNEL) assays were performed to detect cell apoptosis in our *BAD*-overexpressing cell line. In the overexpressing cells, typical apoptotic features were observed on day 5 after transfection (Figure 9). With of time, the number of apoptotic cells in the overexpressing group increased gradually. By 11 days after infection, the deep TUNEL staining areas disappeared and vacuolar structures appeared, indicating that the end of apoptosis. Bright field microscopy showed that the cell morphology of the overexpressing group was markedly different from the other two groups. Furthermore, to distinguish early apoptotic cells from late apoptotic or necrotic cells after overexpression of *BAD* gene in GCs, Annexin V fluorescein isothiocyanate/propidium iodide (FITC/PI) flow cytometric analysis was performed using our *BAD*-overexpressing cell line at days 5, 7, 11, and 13 after infection. During this period, the numbers of living cells in the overexpressing group were almost the lowest among all the three groups at each time point: namely 58.2, 52.1, 53.11, and 57.8%, respectively. The numbers of cells in early apoptosis were also lower than other two groups at 0.58, 0.62, 0.14, and 0.22%, respectively. For cells at late apoptosis, the numbers in the *BAD* overexpressing group were always the highest among the three groups at each time point at 38.9, 45.3, 25.66, and 10.61%, respectively. There were no significant differences in the numbers of necrotic cells among the three groups (Figure 10).

## Discussion

BAD, being a BH3-linked pro-apoptotic protein, has been reported to play an important role in survival signals involved in the mitochondrial cell death machinery (27, 28). Despite extensive research on the role of apoptosis in normal ovarian development and function, limited functional studies have been performed on the role of BAD in GC apoptosis during the transition from a non-breeding to a breeding state among seasonally breeding mammals. There is a close association between apoptotic events and dead granulocytes, which supports the possibility of apoptosis being involved in the induction of follicular atresia (17).

The mammalian estrous cycle involves follicular development and oocyte maturation. A complete estrous cycle can be divided into a follicular phase and a luteal phase. After luteolysis, the levels of P4, which has negative feedback effects on hypothalamic function are reduced, while large quantities of GnRH are secreted by hypothalamus which can stimulate the production of follicle stimulating hormone (FSH) and luteinizing hormone (LH) in the pituitary. FSH and LH changes can promote follicular development and estrogen production, then LH content in the blood increases rapidly and ovulation occurs. As mentioned above, more than 99% of the follicles in the ovary undergo a degenerative process known as atresia induced by apoptosis (21). Sugimoto et al. found that follicular atresia was accompanied by apoptosis of GCs in gilts (22). Thus, the ovary needs to regulate the number, size, and quality of follicles at different stages of development to regulate the timing and quality of female reproduction. The *BAD* gene plays an important role in this process (16).

Here we carried out gene expression profiling during the estrous cycle of two sheep breeds with different seasonality. The complete cDNA sequence of ovine *BAD*, and its distribution in tissues were investigated. *BAD* gene expression could be detected in the uterus, ovary, pituitary, hypothalamus, kidney, lung, spleen, liver, and heart of (28). This distribution is similar to that seen in other mammals such as the human, rat, and yak (9, 23, 28, 27) where the gene is widely expressed in body tissues, particularly the gonads.

Our previous high-throughput sequencing study revealed significant differences in gene expression at different seasons (29), and it was reported that ovarian GCs show changes in function during seasonal reproduction (30). These involve follicular growth factors (FGFs) (31), LH (32), and other proteins that regulate GCs and follicular development (33–35). These changes all involve apoptosis. In mammals, genes encoding the Bcl-2 family of proteins play an important role in promoting or inhibiting apoptosis, and the BH3-linked *BAD* serves as a pro-apoptotic gene.

P4 is a biologically active hormone secreted by the ovaries that can participate in the regulation of the hypothalamic–pituitary–ovarian axis along with E2, and intervene in the ovulation cycle. During the estrous cycle, the level of P4 is the lowest just before ovulation. After ovulation, its secretion began to rise and peaks at the luteal stage. It has negative feedback effects that inhibit its own and E2 secretion. E2 is the most active hormone and is a sign of the start of gonadal function. It is synthesized by the follicular cells and GCs, and its levels are regulated by FSH, LH and steroidgenic genes in the ovary. Under the physiological state, progesterone was produced through the following pathway: cholesterol formed pregnenolone under the action of cholesterol-side-chain cleavage enzyme (P450scc), then pregnenolone in the role of 3β-hydroxysteroid dehydrogenase enzyme (3-βHSD) and CYP11 generated progesterone, which was the precursor of estrogen. Estradiol was produced by the enzymatic action of Cytochrome P450c17-α, aromatase, which coded by gene *Cytochrome 17-*β*HSD*. It can be seen from the results that the expression of Steroidgenic genes in si-*BAD* group and over-*BAD* group showed a similar result, which was in line with the experimental expectation. In si-*BAD* group, the level of progesterone decreased significantly (*P* < 0.05), and the gene *3-*β*HSD, P450scc*, and *CYP11* decreased, and the changes of estradiol were not significant, however, the expression of *17-*β*HSD* was significantly reduced, suggested that estradiol may be regulated by other factors. In over-*BAD* group, the similar regularity was shown: progesterone decreased significantly (*P* < 0.05), and the gene *3-*β*HSD, P450scc*, and *CYP11* decreased significantly, and the changes of estradiol were not significant, the expression of *17-*β*HSD* was significantly different among the three groups. It was suggested that the production of estradiol is affected by other factors.

After RNAi, the level of P4 secreted by our cultured GCs was significantly higher than in the other two groups, but there was no significant difference in E2 concentration. Alterations in P4 concentration might play an important role in the initiation and maintenance of estrous status in sheep (36). Thus, an increase in P4 secretion can be important mainly for inducing estrous behavior and preventing untimely luteolysis during the following estrous cycle (37). Luteolysis is a complex process initiated by a decrease in P4 secretion. The process can last over several estrous cycles in the ovary and has been reported to be caused by apoptosis (21). Moreover, P4 can inhibit the expression of *BAD* to reduce the apoptosis of GCs by binding to the progesterone receptor (progesterone receptor membrane component-1, PGRMC1) (38, 39). Recent studies have shown that increased serum P4 levels can induce estrus and LH secretion, as well as prevent premature lysis of the corpus luteum during the luteal phase (37). Luteolysis has a close relationship with expression of the *BAD* gene and P4 levels in the luteal phase (29). In our study, P4 secreted by GCs was increased significantly after decreased expression of *BAD* using RNAi, while its level was significantly decreased after *BAD* overexpression. Our results suggested that the *BAD* gene might mediate the initiation and maintenance of estrus by regulating the secretion of P4 in GCs.

On the other hand, the concentration of E2 secreted by GCs showed no significant changes after inhibition of *BAD* expression by RNAi, while E2 concentration increased after lentiviral infection. Because testosterone released from theca cells could be changed to P4 under the action of aromatases, the interactions between granulosa and theca cells are important in effecting maximal estrogen biosynthesis (40). Therefore, we speculate that the concentration of E2 secreted by GCs could be affected by coculture with theca cells. However, here we cultured GCs alone, lacking a substrate for estrogen synthesis (androgens), and silencing of the *BAD* gene inhibited apoptosis, resulting in decreased E2 secretion, consistent with a previous study (41, 42). A study on breast cancer cells suggested that E2 is an anti-apoptotic agent whose response is mediated by non-genomic cytoplasmic signal networks that converge on *BAD*, in turn regulating changes in the mitochondrial membrane potential (43). Those results showed that before estrus, the concentration of E2 secretion increased *in vivo* and induced luteolysis; If the follicle is destroyed and the secretion of E2 decreases, corpus luteum degeneration would be prevented. Therefore, it was speculated that the *BAD* gene might change the sheep estrous cycle by regulating the apoptosis of GCs.

In our study there was a significant increase in apoptosis after *BAD* overexpression. Huang et al. explored the role of *BAD* in lung cancer cells, and found that overexpression inhibited the growth of A549 cells *in vitro* and *in vivo* through inhibiting cell proliferation and inducing apoptosis (44). Another study suggested that increased expression of *BAD* enhanced apoptosis and had a negative influence on cell proliferation and tumor growth (45). Overexpression of *BAD* gene can enhance cell cycle progression (46), indicating that it plays an important role in promoting apoptosis and regulating the cell cycle.

The BAD protein plays a regulatory role depending on its phosphorylation status (43, 47, 48). Furthermore, *BAD* interacts with diverse antiapoptotic Bcl-2 members to regulate apoptosis (49), but the exact pathways and mechanisms need further studies.

In conclusion, we found that cell apoptosis was consistent with estrus, which indicates that *BAD* gene expression in the ovary of the sheep changes hormone levels via GC apoptosis, and thereby affects estrous status. The specific pathways need further study.

## Author contributions

XC: collection and/or assembly of data, data analysis and interpretation, manuscript writing. XW: data analysis and interpretation, manuscript writing. LL, XL, RD, XH, and WH: collection and/or assembly of data. XZ: manuscript revising. QL: conception and design, data analysis and interpretation, manuscript writing. MC: conception and design, data analysis and interpretation, final approval of manuscript.

### Conflict of interest statement

The authors declare that the research was conducted in the absence of any commercial or financial relationships that could be construed as a potential conflict of interest.
