# Supplementary material for: Expression and Functional Analysis of the BCL2-Associated Agonist of Cell Death (BAD) Gene in the Sheep Ovary During the Reproductive Cycle
Source: Front Endocrinol (Lausanne). 2018 Sep 19;9:512. doi: 10.3389/fendo.2018.00512 (PMC6156460; doi:10.3389/fendo.2018.00512)
Supplement: Supplementary file 1 [file Presentation_1.PDF]

Supplemental 1 the siRNA screening protocol in detail and the results

To study whether changes in *BAD* expression levels could affect gonadal steroid secretion levels, which are essential for follicular growth and atresia during the estrous cycle, RNAi was performed on primary GCs. First of all, the 3 *BAD*-siRNA molecules were transfected in the follicle GCs cultured in the experiment. The result, as shown in Figure 1, the interference efficiency of siRNA was high. The interference efficiency of *BAD*-siRNA001, 002 and 003 were 92%, 96% and 95%, respectively, when the transfection concentration of siRNA was 30 nM; when siRNA transfection concentration was 50 nM, the interference efficiency of *BAD*-siRNA001, 002 and 003 were 93%, 97% and 90%, respectively. *BAD*-siRNA002 was selected as an interfering molecule to transfect granulosa cells, and the transfection concentration of siRNA was 30 nM.

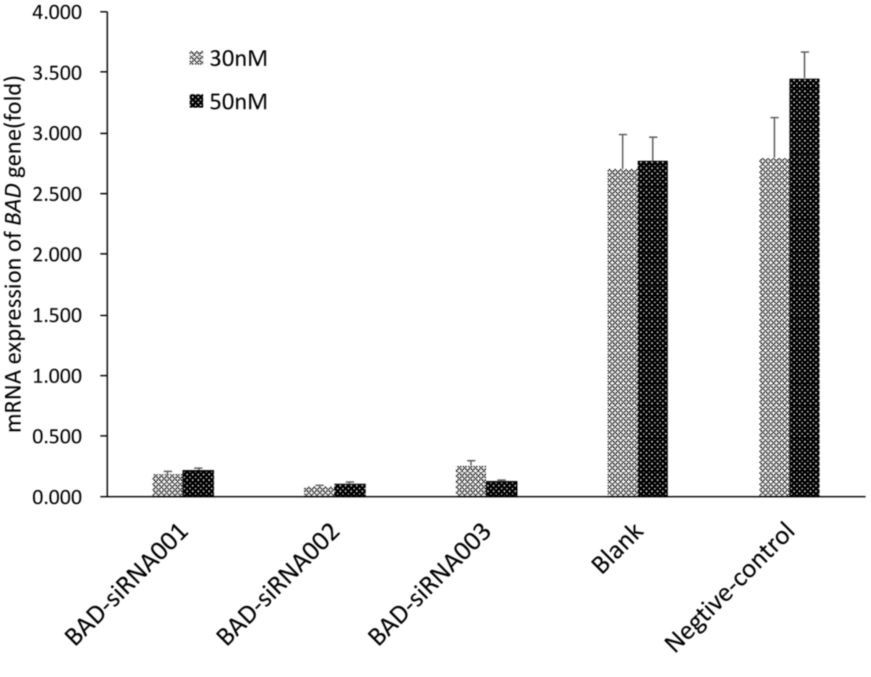

Figure 1 real-time PCR detected the interference efficiency of BAD-siRNA001, 002 and 003 at 30nM and 50nM in vitro
